# Relationship Between the Frequency of Complications After Open-Heart Surgery and Mean Platelet Volume

**DOI:** 10.7759/cureus.102246

**Published:** 2026-01-25

**Authors:** Hakki Kursat Cetin, Sadiye Deniz Ozsoy, Ismail Haberal, Faruk Gencoglu, Ismail Koramaz, Ali Murat Mert

**Affiliations:** 1 Department of Cardiac Surgery, Sisli Etfal Training and Research Hospital, Istanbul, TUR; 2 Department of Cardiothoracic Surgery, Istanbul University, Istanbul, TUR; 3 Department of Cardiovascular Surgery, Istanbul University, Istanbul, TUR

**Keywords:** cardiopulmonary bypass, complication, inflammation biomarkers, mean platelet volume, open cardiac surgery

## Abstract

Objectives: Postoperative complications remain a major cause of morbidity and mortality after open-heart surgery, and early identification of patients at risk is essential for improving outcomes. This study aimed to evaluate the relationship between mean platelet volume (MPV) and postoperative complications following open-heart surgery.

Methods: This retrospective study included 234 patients who underwent open-heart surgery between September 2014 and November 2016 at Istanbul University Cardiology Institute. Patients were divided into two groups based on the presence or absence of postoperative complications. MPV was analyzed as a potential predictive marker of postoperative complications rather than merely an associated parameter. The primary outcome was the occurrence of any postoperative complication. Comparisons between groups were unadjusted for potential confounders such as renal function and comorbidities.

Results: MPV values at the 24th and 48th postoperative hours were significantly higher in patients with complications (p = 0.005, 0.003). Blood urea nitrogen (BUN) and creatinine levels were also significantly higher in the complication group (p = 0.001).

Conclusion: Elevated postoperative MPV levels were associated with a higher frequency of complications after open-heart surgery. These findings suggest that MPV may serve as a useful marker for identifying patients at increased postoperative risk.

## Introduction

Open-heart surgery is a commonly performed procedure for treating various cardiac conditions, including coronary artery disease, valve diseases, and congenital heart defects [1]. Despite advancements in surgical techniques and perioperative care, postoperative complications remain prevalent. These complications, which can range from neurological and renal issues to pulmonary and infectious problems, are exacerbated by factors such as patient age, underlying health conditions, and surgical complexity [2]. The impact of these complications not only affects patient outcomes but also contributes to longer hospital stays and increased healthcare costs, underscoring the need for effective prediction and management strategies for at-risk individuals [3].

One hematological parameter that has drawn attention in the context of cardiovascular diseases and surgeries is the mean platelet volume (MPV). MPV reflects the average size of circulating platelets (PLTs), which play a crucial role in both coagulation and inflammatory processes. Elevated MPV levels have been associated with increased PLT reactivity and thrombotic potential, potentially exacerbating the risk of adverse events following surgical interventions [4]. Recent studies have shown correlations between high MPV levels and complications in conditions like myocardial infarction and unstable angina [5,6]. In the setting of open-heart surgery, fluctuations in MPV may serve as an indicator of inflammatory or thrombotic responses, potentially signaling complications in the early postoperative period. MPV was selected because it directly reflects PLT activation, a key component of both thrombotic and inflammatory responses during and after surgery. Compared with other hematological indices such as PLT distribution width (PDW), neutrophil-to-lymphocyte ratio (NLR), and PLT-to-lymphocyte ratio (PLR), MPV is a simple and reproducible measure that has been linked to poor outcomes in several surgical and cardiovascular settings [7]. Despite this, evidence regarding MPV’s predictive value for postoperative complications after open-heart surgery remains scarce. This study, therefore, aimed to fill this gap by evaluating whether perioperative MPV levels are associated with adverse postoperative outcomes in this population.

This study aimed to investigate the relationship between MPV and postoperative complications in patients undergoing open-heart surgery. By analyzing MPV levels before and after surgery, we aimed to determine whether MPV could serve as a predictive biomarker for adverse outcomes and thereby help guide clinical interventions to improve patient prognosis.

## Materials and methods

This retrospective clinical study included 234 patients who underwent open-heart surgery at Istanbul University's Cardiology Institute between September 2014 and November 2016. Approval for the study was granted by the Cerrahpasa Faculty of Medicine Clinical Research Ethics Committee. Conducted in accordance with the Declaration of Helsinki and Good Clinical Practice guidelines, patient demographic data were collected from hospital records. Participants aged 18 years or older, undergoing elective median sternotomy with on-pump open-heart surgery, were included in the study.

Exclusion criteria were emergency cardiac surgeries, pediatric cases, off-pump surgeries, patients undergoing heart transplants, those with previous cardiac surgeries, and those with a history of major or minor vascular surgery. Patients with incomplete laboratory or clinical data were excluded from the analysis. No imputation was performed for missing variables, and the final analysis included only complete cases. The collected data included demographic features, comorbid conditions (diabetes mellitus, hypertension, hyperlipidemia, and smoking status), and hematologic parameters. Hemogram values (red blood cells (RBCs), white blood cells (WBCs), PLTs, and MPV), serum blood urea nitrogen (BUN), and creatinine levels were measured preoperatively, as well as at 24 and 48 hours postoperatively. MPV was measured from venous blood samples collected in ethylenediaminetetraacetic acid (EDTA)-containing tubes, analyzed within two hours of collection using a Sysmex XN-1000 automated hematology analyzer (Sysmex Corporation, Kobe, Japan) to minimize preanalytical variability.

Patients were divided into two groups based on postoperative complications: Group A (no complications) and Group B (with complications). Complications were classified into cardiac, pulmonary (e.g., pneumonia, pneumothorax, and pleural effusion), neurological (e.g., stroke and transient ischemic attacks), renal (e.g., acute kidney injury), and infections (e.g., sepsis and sternal infections). Postoperative complications were identified and classified according to standardized institutional criteria within the first 30 days after surgery. Complications were reviewed and confirmed by two independent cardiac surgeons based on clinical findings and medical record documentation.

Statistical analysis

Statistical analyses were performed using NCSS (Number Cruncher Statistical System) 2007 (Kaysville, UT, USA). Descriptive statistics were expressed as mean ± standard deviation, median (minimum-maximum), frequency, and percentage. The distribution of continuous variables was assessed using both analytical and graphical methods, including the Kolmogorov-Smirnov test and Q-Q plots. The results of the normality tests guided the choice between parametric (independent samples t-test) and nonparametric (Mann-Whitney U test) analyses for between-group comparisons. For dependent repeated measures, the Friedman test or the Wilcoxon signed-rank test was used as appropriate.

Between-group comparisons included demographics (age, sex, and comorbidities) and laboratory variables (RBC, WBC, PLT, MPV, BUN, and creatinine). Adjustment for multiple comparisons was not applied, as the analyses were exploratory in nature and limited to predefined variables. The study included all eligible patients during the study period (n = 234); therefore, no formal a priori power calculation was performed. However, a post hoc power analysis indicated that the sample size provided 80% power to detect a medium effect size (Cohen’s d = 0.5) for between-group MPV differences at an alpha level of 0.05. Statistical significance was accepted at p < 0.05.

## Results

The demographic analysis of the 234 patients revealed a mean age of 59.3 ± 10.5 years, with a range of 20 to 84 years. Men constituted the majority of the cohort (172 (73.5%)), and 75 (32.1%) of the patients were smokers. Diabetes mellitus was the most common comorbidity (134 (57.3%)), followed by a history of myocardial infarction (103 (44.0%)) and hyperlipidemia (91 (38.9%)). Hypertension was present in 78 (33.3%) of the patients. The echocardiographic ejection fraction (EF) was 53.5% ± 9.3%. Hematological parameters showed a mean RBC count of 4.5 ± 0.6 × 10⁶/µL, mean WBC count of 8.1 ± 2.3 × 10³/µL, and mean PLT count of 244.1 ± 72.1 × 10³/µL. MPV was 8.2 ± 1.0 fL, while BUN and creatinine levels were 16.3 ± 5.6 and 0.9 ± 0.3 mg/dL, respectively (Table 1).

**Table 1:** Demographic characteristics of all cases ECHO: echocardiogram; EF: ejection fraction; RBC: red blood cell; WBC: white blood cell; PLT: platelet; MPV: mean platelet volume; BUN: blood urea nitrogen

Variables	Min-Max	Mean ± standard deviation/n (%)
Age (years)	20.0–84.0	59.3 ± 10.5
Sex, n (%)		
Female		62 (26.5%)
Male		172 (73.5%)
Smoking, n (%)		75 (32.1%)
Diabetes mellitus, n (%)		134 (57.3%)
Myocardial infection, n (%)		103 (44.0%)
Hypertension, n (%)		78 (33.3%)
Hyperlipidemia, n (%)		91 (38.9%)
ECHO EF (%)	20.0–70.0	53.5 ± 9.3
RBC (×10⁶/µL)	2.4–6.2	4.5 ± 0.6
WBC (×10⁶/µL)	3.5–18.0	8.1 ± 2.3
PLT (×10³/µL)	105.0–567.0	244.1 ± 72.1
MPV (fL)	6.1–11.4	8.2 ± 1.0
BUN (mg/dL)	6.0–43.0	16.3 ± 5.6
Creatinine (mg/dL)	0.5–2.5	0.9 ± 0.3

Among the 42 patients who developed postoperative complications (Group B), pulmonary complications were the most common (35.7%), followed by renal (23.8%), cardiac (16.7%), neurological (11.9%), and infectious (11.9%) complications. Patients with postoperative complications (Group B) were significantly older than those without complications (Group A) (64.0 ± 10.0 vs. 58.3 ± 10.3 years, p = 0.001). No multivariable adjustment was performed for age differences. The groups did not differ significantly regarding sex distribution (p = 0.476) or the prevalence of comorbid conditions such as smoking, diabetes mellitus, myocardial infarction, hypertension, and hyperlipidemia (all p > 0.05). Similarly, there was no significant difference in echocardiographic EF (p = 0.453) (Table 2).

**Table 2:** Comparison of demographic characteristics of the groups *Mean ± standard deviation ECHO: echocardiogram; EF: ejection fraction

	Group A (n: 192)	Group B (n: 42)	p-value	Effect sizes (Cramér’s V)
Age (years)*	58.3 ± 10.3	64.0 ± 10.0	0.001	
Sex, n (%)			0.476	0.035
Female	49 (25.5%)	13 (31.0%)		
Male	143 (74.5%)	29 (69.0%)		
Smoking, n (%)	134 (69.8%)	25 (59.5%)	0.173	0.073
Diabetes mellitus, n (%)	110 (57.3%)	24 (57.1%)	0.986	0.001
Myocardial infection, n (%)	82 (42.7%)	21 (50.0%)	0.389	0.045
Hypertension, n (%)	67 (34.9%)	11 (26.2%)	0.278	0.059
Hyperlipidemia, n (%)	76 (39.6%)	15 (35.7%)	0.641	0.019
ECHO EF (%)*	53.7 ± 9.1	52.5 ± 10.2	0.453	

In both groups, postoperative changes were observed in all laboratory parameters. Hematological and biochemical parameters were compared between the groups both at baseline (preoperatively) and at 24 and 48 hours postoperatively to assess perioperative changes. While RBC levels declined over 48 hours in both groups, the differences between the groups were not statistically significant at any time point (p > 0.05). WBC levels showed a progressive increase in both groups, but there were no significant intergroup differences (p > 0.05). PLT levels decreased significantly within each group postoperatively, but intergroup differences were not significant (p > 0.05). MPV values were significantly higher in Group B at 24 and 48 hours postoperatively compared to Group A (p = 0.005 and p = 0.003, respectively). Moreover, BUN and creatinine levels showed a more pronounced increase in Group B compared to Group A at both 24 and 48 hours (p = 0.001 for both parameters) (Table 3).

**Table 3:** Comparison of RBC, WBC, PLT, MPV, BUN, and creatinine values of the groups *Mean ± standard deviation RBC: red blood cell; WBC: white blood cell; PLT: platelet; MPV: mean platelet volume; BUN: blood urea nitrogen

	Group A (n: 192)	Group B (n: 42)	Mean difference (95% CI)	p-value
RBC*				
Preoperative	4.5 ± 0.6	4.5 ± 0.6		0.419
Po 24th hour	3.1 ± 0.4	3.1 ± 0.5		0.220
Po 48th hour	2.9 ± 0.3	3.0 ± 0.3		0.366
Intragroup change p-value	0.001	0.001		
WBC*				
Preoperative	8.1 ± 2.2	8.0 ± 2.6		0.644
Po 24th hour	12.1 ± 3.3	11.6 ± 2.6		0.534
Po 48th hour	14.8 ± 10.4	14.6 ± 3.7		0.152
Intragroup change p-value	0.001	0.001		
PLT*				
Preoperative	243.8 ± 67.9	245.6 ± 89.7		0.552
Po 24th hour	183.3 ± 58.7	164.8 ± 63.0		0.058
Po 48th hour	176.6 ± 63.1	161.3 ± 58.0		0.204
Intragroup change p-value	0.001	0.001		
MPV*				
Preoperative	8.2 ± 0.9	8.3 ± 1.0		0.314
Po 24th hour	8.0 ± 0.9	8.6 ± 1.1	0.6 (0.2–1.0)	0.005
Po 48th hour	8.4 ± 4.8	8.7 ± 1.1	0.3 (0.1–0.6)	0.003
Intragroup change p-value	0.001	0.001		
BUN*				
Preoperative	15.7 ± 5.4	18.9 ± 6.2		0.001
Po 24th hour	18.0 ± 6.4	24.2 ± 9.5	6.2 (3.1–9.3)	0.001
Po 48th hour	22.9 ± 10.9	32.0 ± 14.5	9.1 (5.4–12.8)	0.001
Intragroup change p-value	0.001	0.001		
Creatinine*				
Preoperative	0.9 ± 0.2	1.0 ± 0.3		0.146
Po 24th hour	1.1 ± 0.4	1.4 ± 0.5	0.3 (0.1–0.5)	0.001
Po 48th hour	1.2 ± 0.7	1.7 ± 1.0	0.5 (0.2–0.8)	0.001
Intragroup change p-value	0.001	0.001		

## Discussion

This study aimed to evaluate the relationship between MPV and postoperative complications in patients undergoing open cardiac surgery. MPV is a critical marker of PLT activation and has been associated with thrombotic and inflammatory processes in cardiovascular diseases [8]. Given the significant morbidity and mortality associated with complications following open cardiac surgery, identifying reliable predictive markers is essential for improving patient outcomes [9]. The results of this study contribute to the growing evidence suggesting that elevated MPV levels could serve as an indicator of increased risk for postoperative complications.

The findings demonstrated a statistically significant increase in MPV levels in patients with complications (Group B) at both 24 and 48 hours postoperatively compared to those without complications (Group A). This aligns with previous studies indicating that larger and more active PLTs, as reflected by elevated MPV, contribute to thrombotic events and systemic inflammation [10,11]. The observed elevation in MPV levels may signify heightened PLT turnover and activation in response to surgical stress and inflammatory stimuli in complicated cases. However, the relationship between MPV and postoperative complications observed in this study should be interpreted as associative rather than predictive. Because the analyses were unadjusted for potential confounders such as age and renal function, the findings cannot establish MPV as an independent predictor of adverse outcomes.

Another significant finding was the pronounced rise in BUN and creatinine levels in Group B, suggesting impaired renal function. These changes might result from systemic inflammatory responses, hypoperfusion, or ischemia-reperfusion injury during and after cardiopulmonary bypass. Similar observations have been reported, highlighting that renal dysfunction is a common complication following cardiac surgery and a critical determinant of prognosis [12,13]. Age and renal dysfunction are likely confounding factors in this relationship. Older patients are predisposed to increased PLT activation and systemic inflammatory responses, which may contribute to higher MPV levels. Similarly, the observed elevations in BUN and creatinine levels among patients with complications suggest underlying renal dysfunction, which may influence both PLT morphology and systemic inflammatory states.

The lack of significant differences in RBC, WBC, and PLT levels between the two groups suggests that these parameters, while reflective of systemic responses, may not directly correlate with the occurrence of complications. However, the intragroup changes indicate the profound hematological shifts triggered by open cardiac surgery. The increase in WBC counts postoperatively reflects the systemic inflammatory response, while the decline in PLT levels could be attributed to PLT consumption and sequestration, phenomena frequently described in cardiopulmonary bypass procedures [14].

The findings are consistent with earlier research emphasizing the prognostic value of MPV in cardiovascular outcomes. For instance, studies have shown that elevated MPV is associated with worse outcomes in acute myocardial infarction and coronary artery disease [15,16]. However, its application as a routine marker for predicting surgical outcomes remains underexplored, necessitating further research to validate its utility in clinical settings.

From a clinical perspective, MPV does not replace established risk stratification tools such as the EuroSCORE or Society of Thoracic Surgeons (STS) risk model, but it may provide incremental value as an easily accessible and cost-effective adjunct marker. Alternative explanations for postoperative MPV elevation should also be considered. Factors such as perioperative blood transfusions, cardiopulmonary bypass duration, intraoperative ischemia-reperfusion injury, and perioperative medications (e.g., vasopressors and anti-PLT agents) can independently alter PLT volume and reactivity.

Another important aspect concerns the timing of MPV elevation relative to clinical complication onset. In our cohort, MPV elevations were detected at 24 and 48 hours postoperatively-often preceding the clinical recognition of complications such as renal or pulmonary dysfunction. This temporal pattern suggests that MPV may rise early in the postoperative course, potentially before overt clinical deterioration, supporting its role as a candidate marker for early risk stratification.

These results underscore the potential role of MPV as a biomarker for identifying patients at higher risk for complications postoperatively. Monitoring MPV levels could enable early identification and targeted intervention in at-risk patients, potentially improving surgical outcomes. Additionally, the significant changes in renal parameters suggest the need for stringent renal function monitoring and protective strategies in patients undergoing open cardiac surgery.

This study has several important limitations. Its retrospective and single-center design limits causal inference and generalizability, and the relatively small number of patients with postoperative complications restricts statistical power. Because detailed intraoperative and clinical variables were unavailable, we could not perform multivariable adjustment, leaving the possibility of residual confounding by factors such as age, renal function, transfusion requirement, or cardiopulmonary bypass duration. In addition, variability in MPV measurement due to differences in preanalytical handling or analyzer calibration may have influenced absolute values, although all measurements were performed using a standardized automated hematology analyzer within one hour of sample collection. Despite these constraints, the study’s strengths include the uniform surgical population, standardized perioperative management at a single institution, and serial postoperative MPV monitoring, which together provide a consistent framework for exploring the association between MPV changes and postoperative complications. Future prospective multicenter studies incorporating multivariable modeling are needed to validate these findings and better define their clinical implications.

## Conclusions

In this study, postoperative MPV levels were higher in patients who developed complications following open-heart surgery compared with those who did not. These findings demonstrate an association between elevated MPV and adverse postoperative outcomes but do not establish a causal or predictive relationship. MPV may serve as a supportive biomarker for early postoperative monitoring rather than as an independent predictor of complications. Further prospective multicenter studies with multivariable analyses are warranted to clarify the underlying mechanisms and determine the true prognostic value of MPV in this clinical setting.
